# Pharmaceutical Sovereignty as HIV Justice: Colombia's Dolutegravir License and the Future of ART Access in Latin America

**DOI:** 10.1002/jia2.70137

**Published:** 2026-07-25

**Authors:** Laura M. Cifuentes, Julien B. Brisson, Aarón Zea, Pierre‐Marie David

**Affiliations:** ^1^ Faculty of Pharmacy Université de Montréal Montreal Quebec Canada; ^2^ National Faculty of Public Health “Héctor Abad Gómez” Universidad de Antioquia Medellín Colombia; ^3^ Factor‐Inwentash Faculty of Social Work University of Toronto Toronto Ontario Canada; ^4^ Faculty of Medicine Universidad Nacional de Colombia Bogotá Colombia; ^5^ Más Que Tres Letras Medellín Colombia

1

Access to antiretroviral therapy (ART) remains one of the most pressing challenges in Latin America's HIV response. Despite major biomedical advances, persistent inequalities in affordability, availability and regional production of ART continue to impede progress towards the UNAIDS 95‐95‐95 targets [1]. Latin America has an estimated 2.3 million people living with HIV, of whom approximately 73% are receiving ART and only 67% have achieved viral suppression [1]. Access remains closely tied to health systems financing, as ART costs constitute a substantial share of national HIV budgets. In Colombia, for instance, HIV‐related expenditures reached USD 194.6 million in 2022 [2]. Recent international HIV funding cuts are projected to exacerbate structural vulnerabilities, increasing the risk of ART access interruptions and inequities across the region [3].

These vulnerabilities stem from systemic barriers—high drug prices, restrictive patent and procurement regimes, and limited domestic manufacturing—constraining equitable HIV treatment in the region [4]. At the same time, HIV distribution in Latin America remains uneven: gay, bisexual and other men who have sex with men, transgender women, sex workers, migrants and people in prisons or other closed settings face disproportionately high acquisition rates and unmet treatment needs, exacerbated by stigma, discrimination and criminalization [5, 6]. When those most affected are marginalized or deprioritized, chronic underinvestment perpetuates exclusion. Ensuring reliable, affordable ART access is not merely logistical but a matter of social justice, requiring states to uphold the rights and dignity of populations pushed to the margins of public health priorities.

Against this backdrop, Colombia's April 2024 compulsory license for government use of dolutegravir represents a pivotal shift in regional HIV governance [7]. It reflects a deliberate political move towards pharmaceutical sovereignty: the ability of states to ensure equitable, sustainable access to essential medicines through legal, regulatory and manufacturing capacity, and public‐interest decision‐making [8].

By reducing the annual cost per person of a WHO‐recommended first‐line antiretroviral drug by 96%—from approximately USD 1160 to USD 42 [9]—the compulsory license simultaneously disrupts pricing inequities and affirms the primacy of the right to health over commercial claims. Although Brazil's 2007 compulsory license for efavirenz set a precedent, no Latin American country had taken similar measures in nearly two decades. Thus, Colombia's action goes beyond policy innovation: it declares ART access a fundamental entitlement rather than a market‐dependent privilege, underscoring that equitable ART access is inseparable from broader struggles for justice and the right to health in Latin America.

Colombia invoked the “government use” clause of Andean Decision 486, a regional intellectual property framework governing patents across the Andean Community (Bolivia, Colombia, Ecuador and Peru). This mechanism is supported under the Agreement on Trade‐Related Aspects of Intellectual Property Rights (TRIPS) and reaffirmed in the Doha Declaration, which states that public health takes precedence over patent protections. By activating it, Colombia demonstrated what other countries in the region have lacked: legal readiness with political will. In Colombia, previous civil society petitions—such as for lopinavir/ritonavir in 2008 or direct‐acting antivirals for hepatitis C in 2015—resulted in price reductions as the government's main strategy for expanding access. However, this approach reinforced dependence on multinational suppliers. In contrast, the dolutegravir license marks a shift: the state is no longer a passive negotiator but an active actor producing a generic version of this essential drug [10]. Enabled by years of civil society advocacy—including efforts by community organizations such as *Más Que Tres Letras* to challenge the patent on dolutegravir [11, 12]—this shift demonstrates how community power and state capacity can advance justice‐oriented HIV governance.

The significance of Colombia's decision extends beyond its borders. Many Latin American countries remain structurally disadvantaged within global pharmaceutical markets: reliant on external supply chains, constrained by small procurement volumes and excluded from voluntary licensing for next‐generation antiretrovirals. The case of lenacapavir, a long‐acting injectable antiretroviral, illustrates this exclusion. Despite its relevance for populations facing Antiretroviral therapy (ARV) adherence barriers, access remains unavailable in most Latin American countries, and TRIPS flexibilities are limited by Gilead's non‐exclusive bilateral licensing strategy [13]. This pattern exposes the limits of current licensing frameworks and reveals how market‐based models reproduce inequities, including coercive practices that condition access to preventive medicines, such as for HIV, on natural resource negotiations [14].

Quite differently, the voluntary licence of dolutegravir obtained via the Unitaid‐supported Medicines Patent Pool allows countries outside the voluntary licence territory to purchase generics from licensees as long as the patent is not in force, as in Chile and Peru, or if it has been revoked through compulsory licensing, as in Colombia. Hence, Colombia's use of TRIPS flexibilities, therefore, opens a pathway for regionwide transformation. With coordinated action, institutions such as the Pan American Health Organization (PAHO) Strategic Fund, MERCOSUR (South American trade block), the Andean Community and CELAC (Community of Latin American and Caribbean States) could serve as powerful platforms for pooled procurement, shared legal expertise, technology transfer and regional manufacturing capacity. In practice, access to dolutegravir in Colombia has already reflected this type of regional collaboration via the Pan American Health Organization Revolving Funds mechanism, through which the country has been able to make its first centralized purchases of tenofovir/lamivudine/dolutegravir at substantially lower prices [15]. Pharmaceutical sovereignty is, therefore, not an individual national project but a collective strategy for regional solidarity and HIV treatment justice. This is especially relevant beyond dolutegravir itself, as limited access to antiretrovirals, including lenacapavir, risks widening disparities in HIV prevention and care.

However, lowering cost is a foundation, not an endpoint. Long‐acting regimens could transform adherence, yet without regional procurement and bargaining power, many Latin American countries risk exclusion from these innovations. Colombia's licensing decision creates political space to adopt emerging technologies and align pharmaceutical governance with equitable access. This was reinforced on 27 April 2026, when the Andean Tribunal ruled in favour of Colombia in a case brought by ViiV Healthcare and Shionogi & Co., Ltd., upholding Colombias's compulsory license and finding no violation of Andean industrial property law [16]. This ruling legally consolidates Colombia's compulsory license, enabling generic dolutegravir access domestically despite patent protection elsewhere [17].

Colombia's decision on dolutegravir is both a national achievement and a regional call to action. It demonstrates that legal preparedness, mobilization and political courage can realign HIV governance with justice and sovereignty. In a region marked by inequality and exclusionary politics, asserting public authority over essential medicines is both an ethical imperative and a practical way to build capacity. Without strengthening legal and manufacturing capacities, Latin America risks being locked out of the next era of ARV innovations. Colombia has shown that a different future is possible. The region must now advance coordinated strategies centred on justice in HIV treatment access.

## Author Contributions

LMC conceptualized the manuscript, conducted the information search and drafted the text. JBB contributed to conceptualization, reviewed and edited the manuscript, and handled translation. P‐MD contributed to conceptualization and provided critical review and editorial input. AZ helped strengthen the argument through regional expertise on HIV.

## Conflicts of Interest

The authors have no conflicts of interest to declare.

## Data Availability

All data generated or analyzed during this study are included in this article.
